# Supplementary figures and images for: A decision tree model to predict liver cirrhosis in hepatocellular carcinoma patients: a retrospective study
Source: PeerJ. 2023 Aug 24;11:e15950. doi: 10.7717/peerj.15950 (PMC10460570; doi:10.7717/peerj.15950)

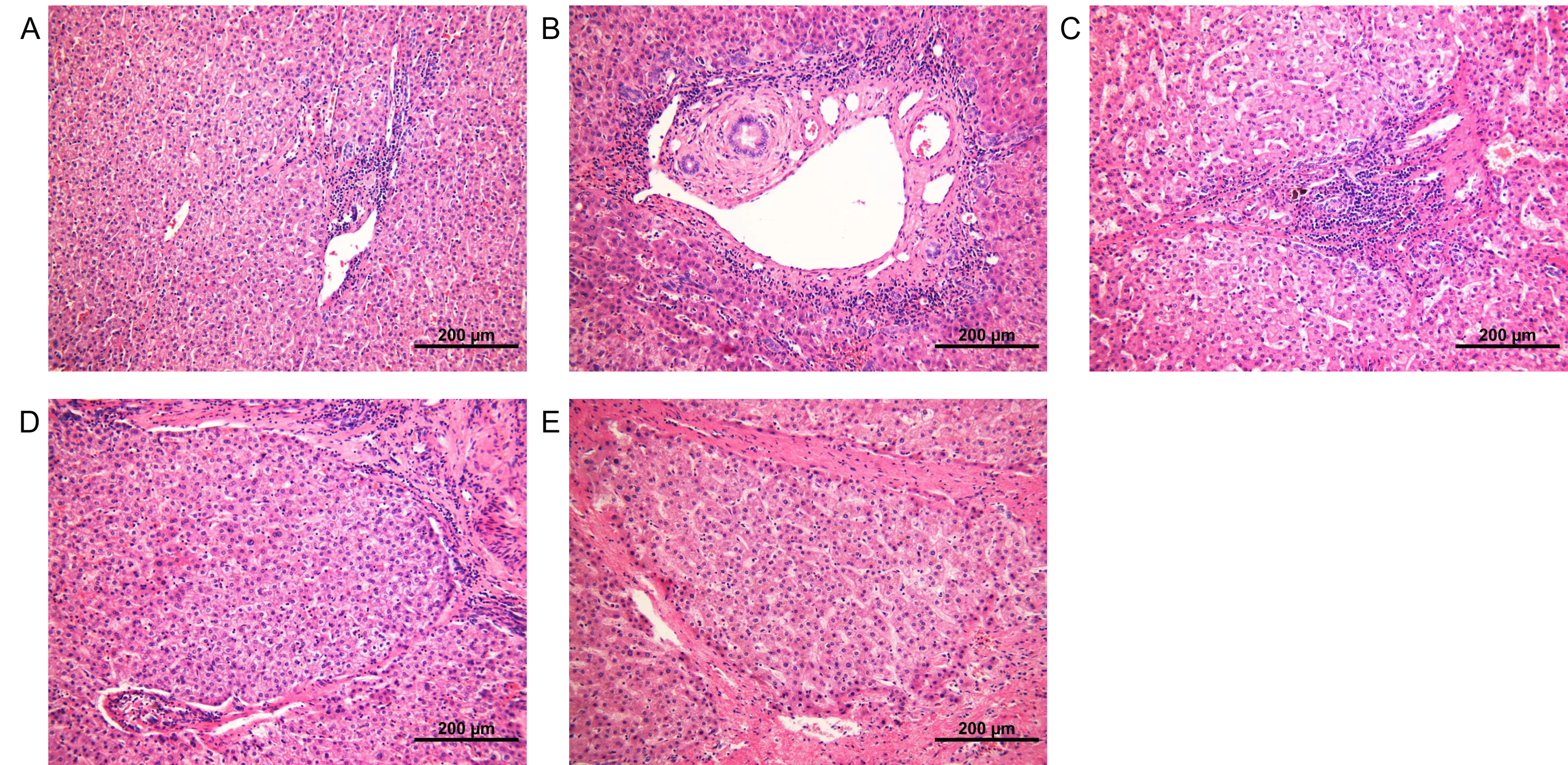

Supplement: Supplemental Information 1 [file peerj-11-15950-s001.png]

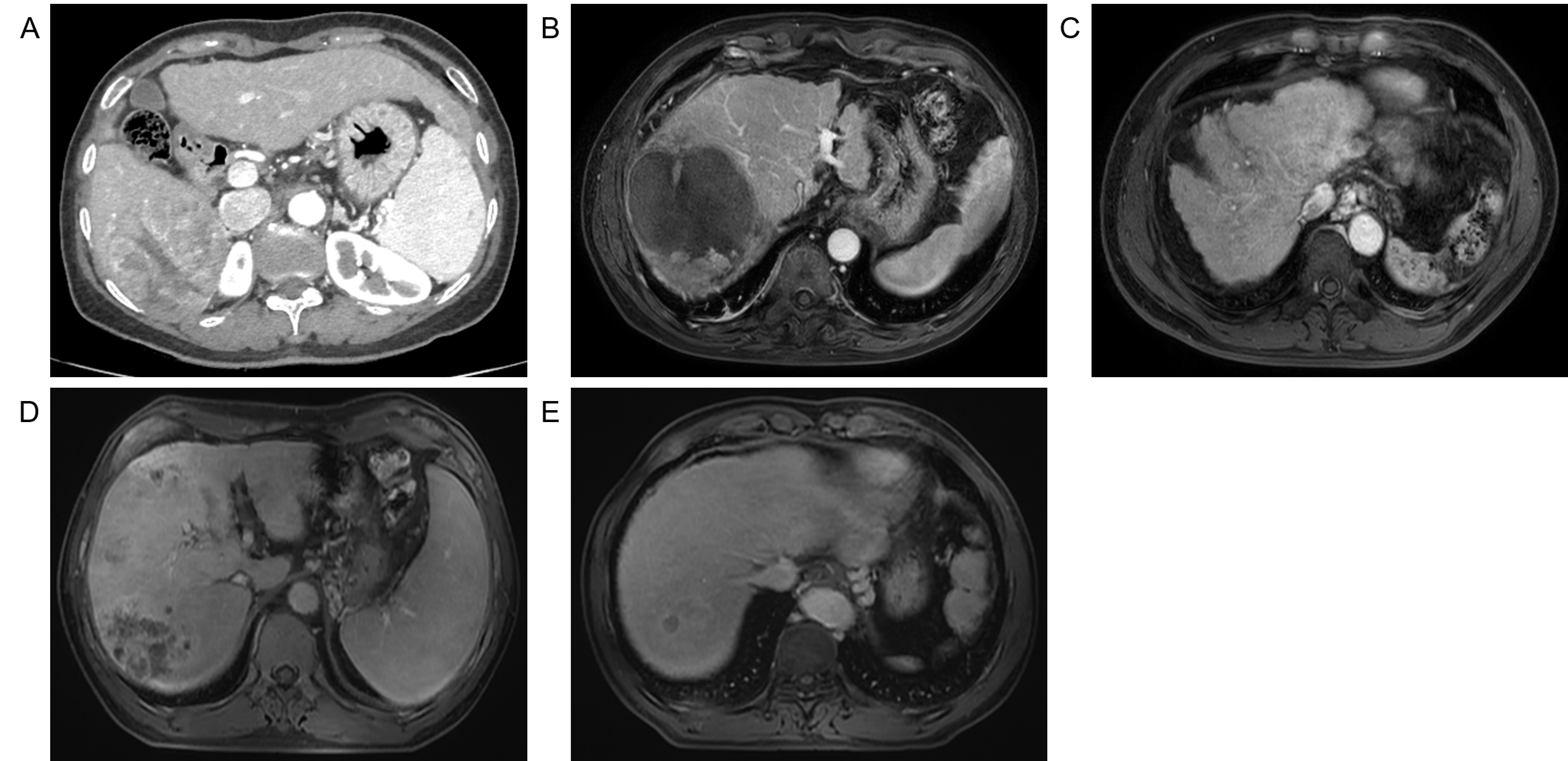

Supplement: Supplemental Information 2 [file peerj-11-15950-s002.png]

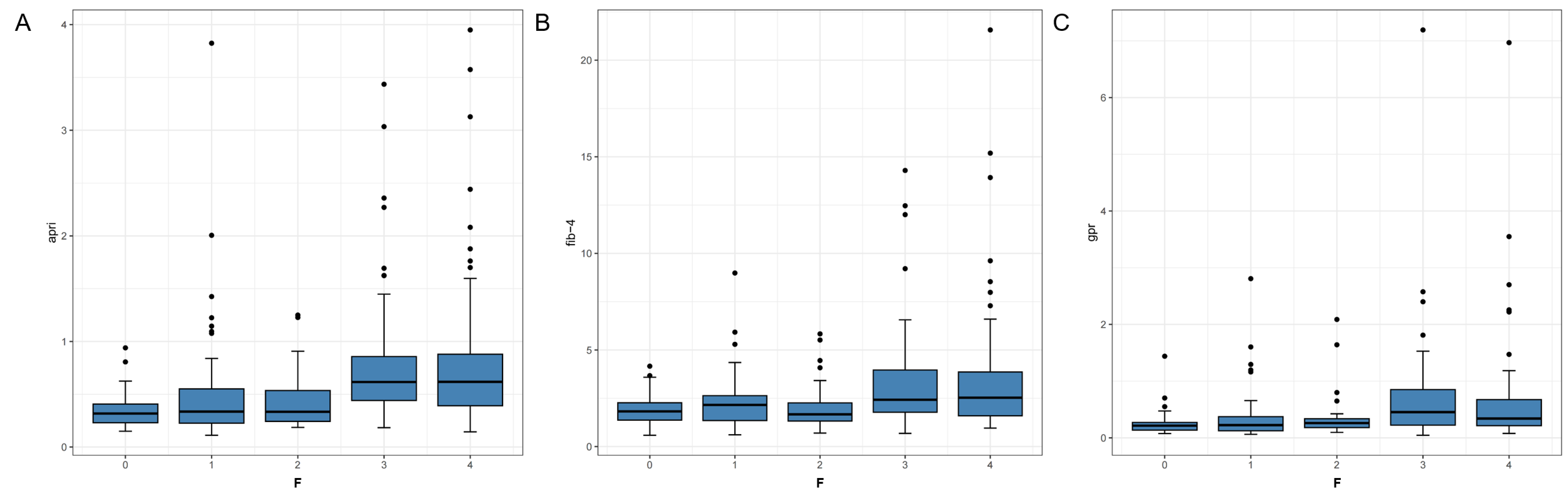

Supplement: Supplemental Information 3 [file peerj-11-15950-s003.png]

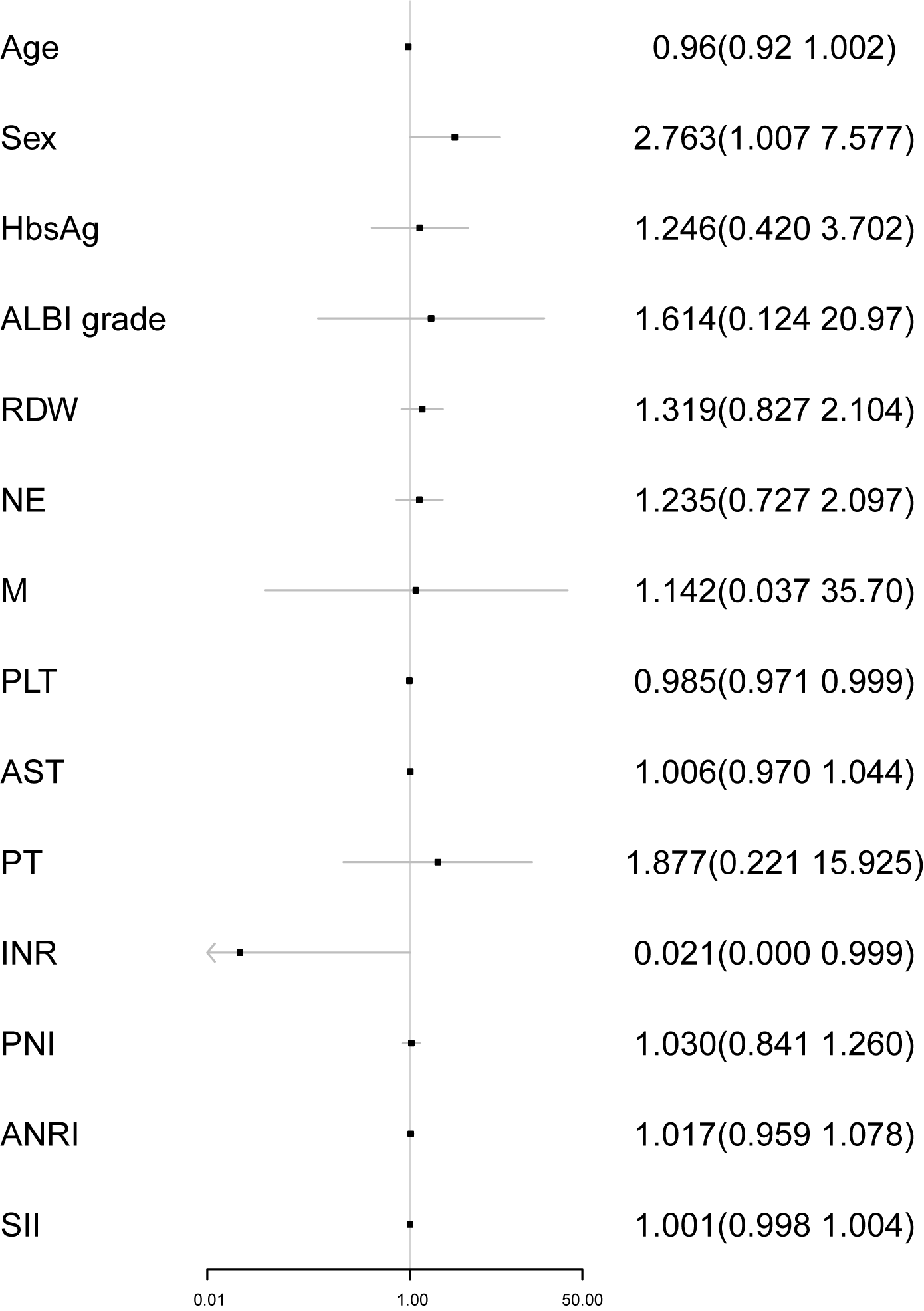

Supplement: Supplemental Information 4 [file peerj-11-15950-s004.png]
