# Supplementary material for: A decision tree model to predict liver cirrhosis in hepatocellular carcinoma patients: a retrospective study
Source: PeerJ. 2023 Aug 24;11:e15950. doi: 10.7717/peerj.15950 (PMC10460570; doi:10.7717/peerj.15950)
Supplement: Supplemental Information 6 [file peerj-11-15950-s006.docx]

1. **Sex:** 1: male; 2: female
2. **HbsAg:** 1: positive; 0: negative
3. **HCVAb:** 1: positive; 0: negative
4. **ALBI grade:** 1: ≤ -2.60; 2: more than -2.60 to ≤ -1.39; 3: > -1.39
5. **INR group:** 1: ≤ 1; 2: > 1
6. **Tumor number:** 1: Multiple; 0: Solitary
7. **Tumor size group:** 1: ≤ 5 cm; 2: > 5 cm
8. **Cirrhosis:** 1: Present; 0: Absent

Above is an explanation of the categorical variables in the raw data.
